# Compassionate Use of Yuanjiang Decoction, a Traditional Chinese Medicinal Prescription, for Symptomatic Bradyarrhythmia

**DOI:** 10.3389/fphar.2022.764930

**Published:** 2022-04-11

**Authors:** Zhang Wan-Tong, Zhu Bao-Chen, Liu Zhao, Wang Xu-Jie, Gao Rui, Xiao Ning, Tang Wei, Wu Yu-Fei, Phoebe Miles, Weng Wei-Liang, Lin Hao-Xiang, Li Qiu-Yan

**Affiliations:** ^1^ Institute of Clinical Pharmacology, Xiyuan Hospital, China Academy of Chinese Medical Sciences, Beijing, China; ^2^ National Clinical Research Center for Chinese Medicine Cardiology, Beijing, China; ^3^ NMPA Key Laboratory for Clinical Research and Evaluation of Traditional Chinese Medicine, Beijing, China; ^4^ Department of Pharmacy, Dongzhimen Hospital, Beijing University of Chinese Medicine, Beijing, China; ^5^ Tobacco Medicine and Tobacco Cessation Center, China-Japan Friendship Hospital, Beijing, China; ^6^ Faculty of Humanities and Social Sciences, University of Nottingham, Ningbo, China; ^7^ Department of Social Medicine and Health Education, School of Public Health, Peking University Health Science Center, Beijing, China; ^8^ Stroke Center, Xiyuan Hospital, China Academy of Chinese Medical Sciences, Beijing, China

**Keywords:** bradyarrhythmia, compassionate use, Yuanjiang decoction, traditional Chinese medicinal prescription, prospective pilot trial

## Abstract

**Background:** No effective medication is available for symptomatic bradyarrhythmia, particularly in low socioeconomic status (SES) population.

**Objective:** To explore the safety and efficacy of Yuanjiang decoction, a traditional Chinese medicinal prescription, for symptomatic bradyarrhythmia on a compassionate-use basis.

**Methods:** This compassionate-use study was conducted in Beijing, China between January 2019 and January 2020. Eligible participants were recruited and treated with Yuanjiang decoction (composed of 6 Chinese herbal medicines), 200 ml twice daily for 16 weeks. Analyses were done with the intention-to-treat (ITT) approach. The primary outcome measure was the proportion of participants who achieved a favorable treatment outcome at 16 weeks.

**Results:** As of January 2020, 184 patients were included. After 16-weeks treatment, 12 participants were lost to contact while 21 participants were terminated from this study, with a drop-out rate of 17.93%. The most common treatment-related adverse events were xerostomia (6.52%), constipation (6.45%) and sleepiness (3.26%). The proportion of participants with favorable treatment outcome was 65.22% at 4 weeks, 59.78% at 8 weeks (OR: 1.11, 95% CI: 0.71–1.73), 61.41% at 12 weeks (OR: 1.16, 95% CI: 0.92–1.45) and 60.87% at 16 weeks (OR: 1.15, 95% CI: 0.98–1.35). In the multifactor regression analysis, the favorable treatment outcome at 16 weeks was significantly associated with completing at least 8 weeks treatment (OR: 2.053, 95% CI: 1.064–3.560), while unfavorable treatment outcome was significantly associated with an atrioventricular block (OR: 0.255, 95% CI: 0.083–0.784), current smoking (OR: 0.343, 95% CI: 0.027–0.487), and syncope in the month before treatment (OR: 0.321, 95%CI: 0.114–0.904).

**Conclusion:** This compassionate-use study showed encouraging outcomes of treatment with Yuanjiang decoction, without serious adverse events. This study identified several key factors that may affect outcomes. These findings helped inform the design and assess the feasibility of a large-scale randomized clinical trial.

## Introduction

Bradyarrhythmia is a severe cardiac arrhythmia in clinical practice, characterized by slow heart rate and hemodynamic disorders which can result in blood insufficiency. The causes of bradyarrhythmia mainly include sinus-node dysfunction, atrioventricular-conduction disturbances and intraventricular block (Honarbakhsh et al., 2018). In severe cases, bradyarrhythmia can cause circulatory disturbance and endanger life.

The prevalence of bradyarrhythmia is rapidly increasing, owing to an ageing population (Ma et al., 2020). Despite its significance, only limited pharmaceutical interventions, such as atropine and isoproterenol, can be used to increase heart rate in the short-term. More importantly, there are no approved medications currently available for long-term use (Neumar et al., 2010). In the absence of proven effective medication, clinically significant bradyarrhythmia with typical symptoms requires pacemaker implantation (Epstein et al., 2013). The number of pacemaker implantations in China has greatly increased from 25,000 in 2007 to 73,000 in 2016 (Hua and Zhang, 2018). However, the quality of life in patients with pacemaker implantation is severely affected due to a high incidence of complications such as heart failure and infection (Johansen et al., 2011; Gillam et al., 2018), life-long follow-up demand (Mond and Proclemer, 2011) and its high cost (Clémenty et al., 2018). Therefore, many individuals refuse or cannot afford to pacemaker implantation, especially in low socioeconomic status (SES) population (Clémenty et al., 2019). This population is at the greatest risk for severe complications, and there is an urgent need to find out an alternative option for them.

Traditional Chinese medicine has a long history of treating bradyarrhythmia, which may have begun as early as 2,000 years ago (Chan, 2005). In recent years, several clinical trials demonstrated TCM, including Shenxian-Shengmai oral solution (Gao et al., 2019), Shensong Yangxin capsule (Liu et al., 2014), and Xinbao pill (Hu et al., 2020) has potential therapeutic effects for patients with bradyarrhythmia, without the need for pacemaker implantation. To date, no effective treatment is available for those who need pacemaker implantation but cannot afford the high cost.

As such, to provide an alternative option for symptomatic bradyarrhythmia patients requiring pacemaker implantation, we proposed an innovative herb formula, Yuanjiang decoction. This proposal is based upon the herb properties (Liu et al., 2018), recommendations from existing studies and guidelines (Yang et al., 1992; Wen et al., 2007; Cao et al., 2020), and our clinical practice experience (Li et al., 2017). This decoction consists of 6 herbs, including *Alpinia officinarum Hance* (Family: Zingiberaceae, Plant part: *Rhizome)*, 12 g; *Zingiber officinale Roscoe* (Family: *Zingiberaceae*, Plant part: *Rhizome*), 6 g; *Cinnamomum verum J.Presl* (Family: Lauraceae, Plant part: *Cortex*), 6 g; *Piper longum L*. (Family: *Piperaceae*, Plant part: *Fructus*), 12 g; *Corydalis yanhusuo* (Family: *Papaveraceae*, Plant part: *Rhizome*), 15 g; *Curcuma aromatica Salisb*. (Family: *Zingiberaceae*, Plant part: *Radix*), 12 g. Yuanjiang decoction appears to have a favorable clinical safety profile, as reported based on our clinical practice experience (Liu and Weng, 2020). Moreover, previous studies have shown potential therapeutic efficacy in non-clinical models (Zhang et al., 2018). In this report, we describe the outcomes in a cohort of patients with low SES treated for symptomatic bradyarrhythmia with Yuanjiang decoction on a compassionate-use basis, in order to inform the design and assess the feasibility of a large-scale randomized clinical trial.

## Methods

### Study Design

We conducted a prospective pilot trial of compassionate use of Yuanjiang decoction for symptomatic bradyarrhythmia among low SES patients between January 2019 and January 2020 in Beijing, China. The protocol of this study is shown in Supplementary Appendix SA. This study was reviewed and approved by the institutional review board of Xiyuan Hospital of China Academy of Chinese Medical Sciences before the start of the study (No. 2018XLA035-2) and registered in Chinese Clinical Trial Registry (No. ChiCTR-1800018464).

As this was a compassionate-use study, the Xiyuan Hospital of China Academy of Chinese Medical Sciences established a specific Data and Safety Monitoring Board (DSMB) to monitor the study once per month and received summaries of study conduct, safety, and efficacy.

### Participants

Eligible participants were recruited via trial sites, local newspapers, community events, websites, and recommendations from other medical institutions. Participants were included if they met all the following inclusion criteria: 1) aged 18–90 years; 2) documented symptomatic bradyarrhythmia; 3) Class I indications of permanent pacemaker implantation; 4) low SES and cannot afford pacemaker implantation due to inadequate finance; 5) voluntary participation in this trial and able to provide informed consent. Also, females were required to have a negative urine pregnancy test before starting treatment.

Participants were excluded if they met any of the following criteria: 1) pregnant or lactating; 2) allergic predisposition and drug allergy to the 6 herbs in the decoction; 3) comorbidities including severe liver disease, severe renal dysfunction, persistent atrial fibrillation, acute myocardial infarction, stroke, hematopoietic system abnormalities and mental illness in the past 3 months; 4) drug-induced transient secondary bradyarrhythmia; 5) athletes with low heart rate but normal sinoatrial node function, without bradyarrhythmia-related symptoms; 6) participation in other clinical trials in the last month; 7) taking any Chinese medicinal herbs in the last month.

The trial adhered to the Declaration of Helsinki. All participants signed informed consent and received financial compensation for trial participation time and travel expenses.

### Compassionate Use and Ethical Consideration

Compassionate use is a treatment option that allows for exceptional use of unapproved drugs to treat patients with life-threatening or severe disease (Zhang et al., 2018). For the protection of the participants, at the request of the DSMB, the analysis of the primary outcome was completed once a month. The participants who showed syncope during the treatment with heart rate less than 30 bpm were terminated from this study and referred for further treatment, if appropriate.

Another important ethical consideration was that currently, as there are no alternative approved medications for this condition, there cannot be a non-treatment control group for apparent ethical reasons. Therefore, we did not define a control group in this study.

### Procedures

All participants received 16 weeks of Yuanjiang decoction, self-administered, 200 ml orally, twice per day and supportive therapy was provided at the discretion of the clinicians.

**FIGURE 1:**
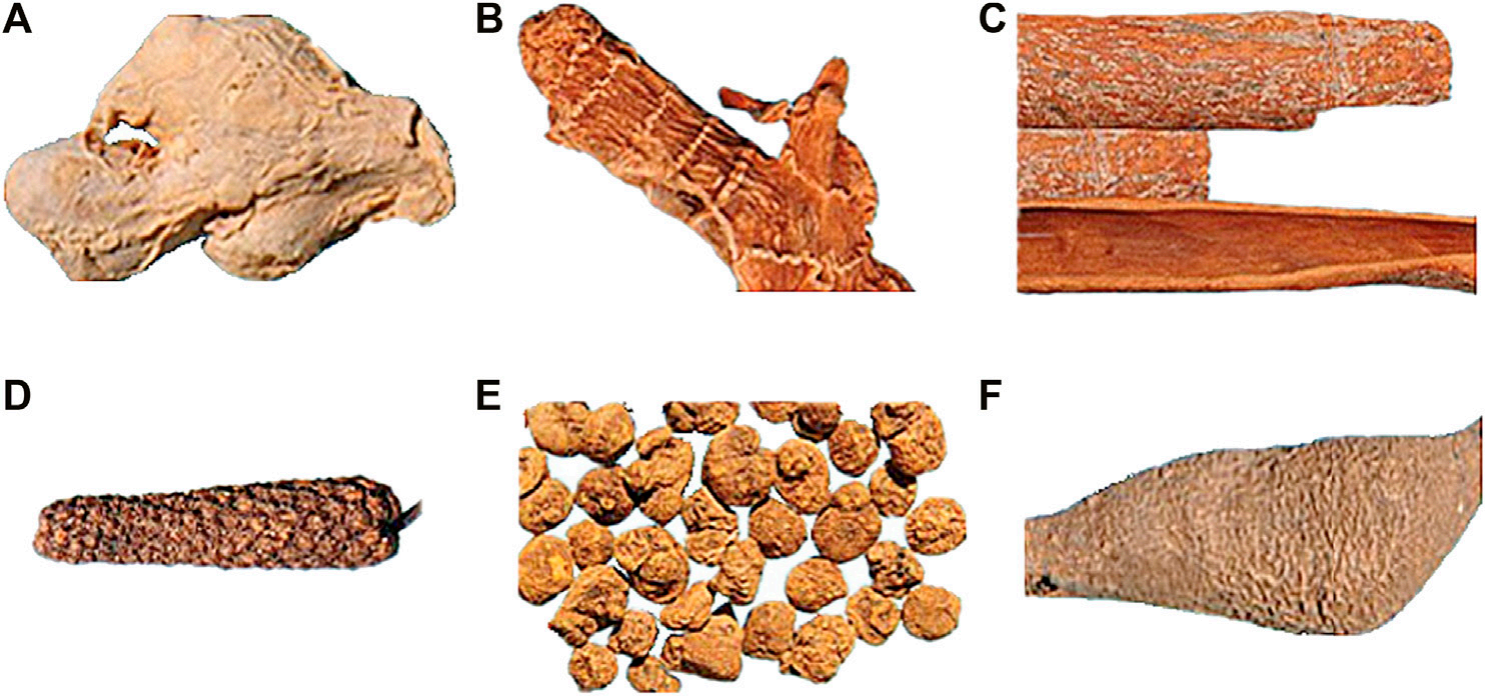
Example of 6 herbs of Yuanjiang decoction. **(A)**, *Zingiberis rhizoma*; **(B)**, *Alpiniae officinarum rhizoma*; **(C)**, *Cinnamomi cortex*; **(D)**, *Piperis longi fructus*; **(E)**, *Corydalis rhizoma*; **(F)**, *Curcumae radix*.

This decoction consists of 6 herbs: including Alpinia officinarum Hance (Family: *Zingiberaceae*, Plant part: *Rhizome*), 12 g; *Zingiber officinale Roscoe* (Family: *Zingiberaceae*, Plant part: *Rhizome*), 6 g; *Cinnamomum verum J.Presl* (Family: *Lauraceae*, Plant part: *Cortex*), 6 g; Piper *longum L.* (Family: *Piperaceae*, Plant part: *Fructus*), 12 g; *Corydalis yanhusuo* (Family: *Papaveraceae*, Plant part: *Rhizome*), 15 g; *Curcuma aromatica Salisb*. (Family: *Zingiberaceae*, Plant part: *Radix*), 12 g (Figure 1).

**FIGURE 2:**
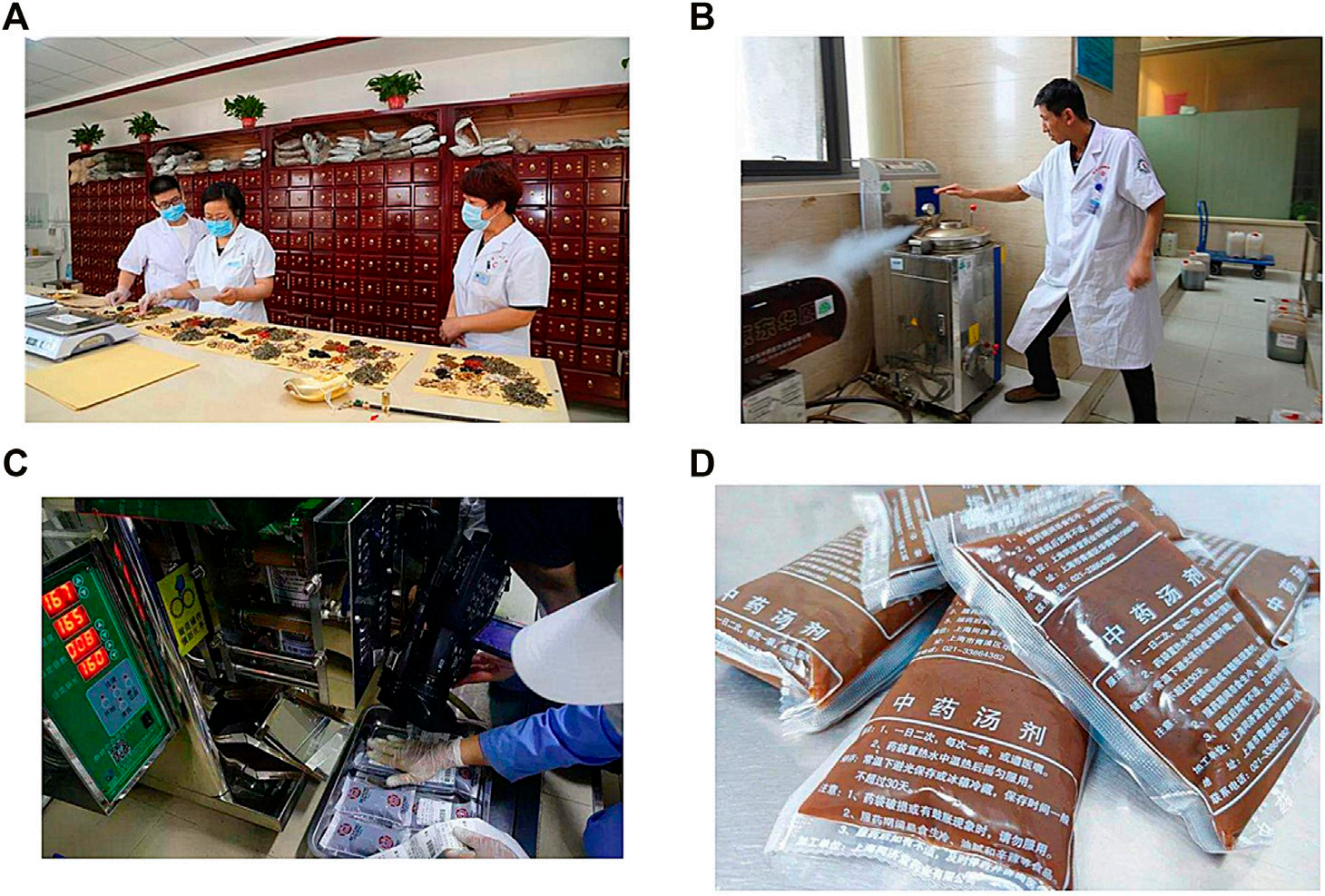
The procedure for preparing a decoction **(A)**, herbs preparation; **(B)**, machine decocting; **(C)**, decoction packing; **(D)**, decoction packages distributed to participants.

The procedure for preparing decoction is shown in Figure 2. In brief, a specialist pharmacist was responsible for decoction management and quality control. Before decoction preparation, all herbs were tested for heavy metals, microbial contamination, and residual pesticides. All the herbs were weighed precisely and placed in non-woven decocting bag (40*40 cm), then they were transferred into an automatic decocting machine (Beijing Donghuayuan Medical Equipment Co. Ltd., YJD20-GL). Before decocting, the herbs were immersed in water (drug-solvent ratio 1:15) for 30 min. Then they were decocted under 110° (Celsius) and 150 kPa for 30 min and concentrate to 400 ml. Finally, the decoctions were automatically packed with a specification of 200 ml. Our study comply with the best practice guidelines of the leading journals for pharmacological studies on natural products (Heinrich et al., 2020).

Participants were instructed to drink the Yuanjiang decoctions 30 min after breakfast and dinner, respectively, and not to take any other drugs, tea or coffee within half an hour before and after taking the decoction to avoid possible reactions with the Chinese medicine ingredients.

For the present study, we examined the effects of Yuanjiang decoction at 4, 8, 12, and 16 weeks into treatment.

### Outcomes

The primary outcome was the proportion of participants who achieved a favorable treatment outcome at 16 weeks. The favorable treatment outcome was the improvement of bradyarrhythmia-related symptoms. This was defined as either or both, a minimum increase of 5 bpm in heart rate, or a minimum heart rate of more than 40 bpm (Zhang et al., 2019). The bradyarrhythmia-related symptoms were assessed using self-completed visual analogue scales (VAS). It assessed a specific symptom identified by the patient as being the most troublesome during prior therapy (symptom-specific VAS score). The patient-specific symptoms included manifestations of syncope or near syncope, transient dizziness or lightheadedness, or confessional states resulting from cerebral hypoperfusion attributable to slow heart rate. Fatigue, exercise intolerance may also result from bradyarrhythmia. The stem question for the symptom-specific VAS was, “How severe was your [X] today?” in which “X” was replaced with the patient’s specific symptom. For VAS questions, the patient responded by making a mark between 0 mm (no symptoms) and 100 mm (maximum intensity for the symptom in question) on a horizontal line. A difference of at least 13 mm in the VAS pain score was considered to be clinically significant (Breivik et al., 2008).

For this study, the definition of Class I indications of permanent pacemaker implantation was based on the ACC/AHA/HRS 2008 Guidelines (Epstein et al., 2008). The definitions of sinus node dysfunction, sinus pause, sinoatrial exit block and atrioventricular block were based on 2018 ACC/AHA/HRS Guideline (Kusumoto et al., 2018). The low SES was defined as monthly income less than 1600 China Yuan (CNY), approximately 230 US dollars. The definition of myocarditis was based on the Current State of Knowledge on Aetiology, Diagnosis, Management, and Therapy of Myocarditis (Caforio et al., 2013). The definition of coronary artery disease was based on the 2016 ACC/AHA Guideline (Levine et al., 2016). The definition of hypertension was based on the 2018 ESC/ESH Guideline (Emrich et al., 2019). The definition of Paroxysmal AF and atrial premature complexes was based on the 2015 ACC/AHA/HRS Guideline (Page et al., 2015). The definition of premature ventricular contractions was based on the 2017 AHA/ACC/HRS Guideline (Al-Khatib et al., 2017). The definition of diabetes mellitus was based on the Update on Prevention of Cardiovascular Disease in Adults with Type 2 Diabetes Mellitus in Light of Recent Evidence (Fox et al., 2015). The definition of current smoking was having smoked 100 cigarettes in one’s lifetime and currently smoking cigarettes (George et al., 2019).

### Statistical Analysis

Based on our clinical practice experience (Li et al., 2017), the proportion of participants who achieved a favorable treatment outcome at 16 weeks was estimated to be 60%. A sample size of 180 participants was calculated to provide 80% power, assuming a 20% drop-out.

Intent-to-treat (ITT) approach was applied in this analysis, and the participants who were lost to contact or terminated from the study were considered to be unfavorable treatment outcome. The measurement data were presented as means±SDs. The t-test was used for comparisons which met Gaussian distribution and homogeneity of variance, whereas the nonparametric test was used for comparisons which did not meet homogeneity of variance. The chi-square test was used for numeral data. The logistic regression analysis was used to calculate the relationship between potential influencing factors and efficacy, represented with OR value at the 95% confidence interval. The Kaplan-Meier curve was used for analyses of optimal treatment time. SPSS 19.0 statistical software (SPSS, Inc.) was used for statistical analysis. *p* < 0.05 was regarded as statistical significance.

The authors had no access to information that could identify individual participants during or after data collection.

### Role of the Funding

The funders of the study had no role in the study design, data collection, data analysis, data interpretation, or writing of the report. All authors had full access to the data, participated in data analysis and manuscript development, and gave final approval of the manuscript.

## Results

**FIGURE 3:**
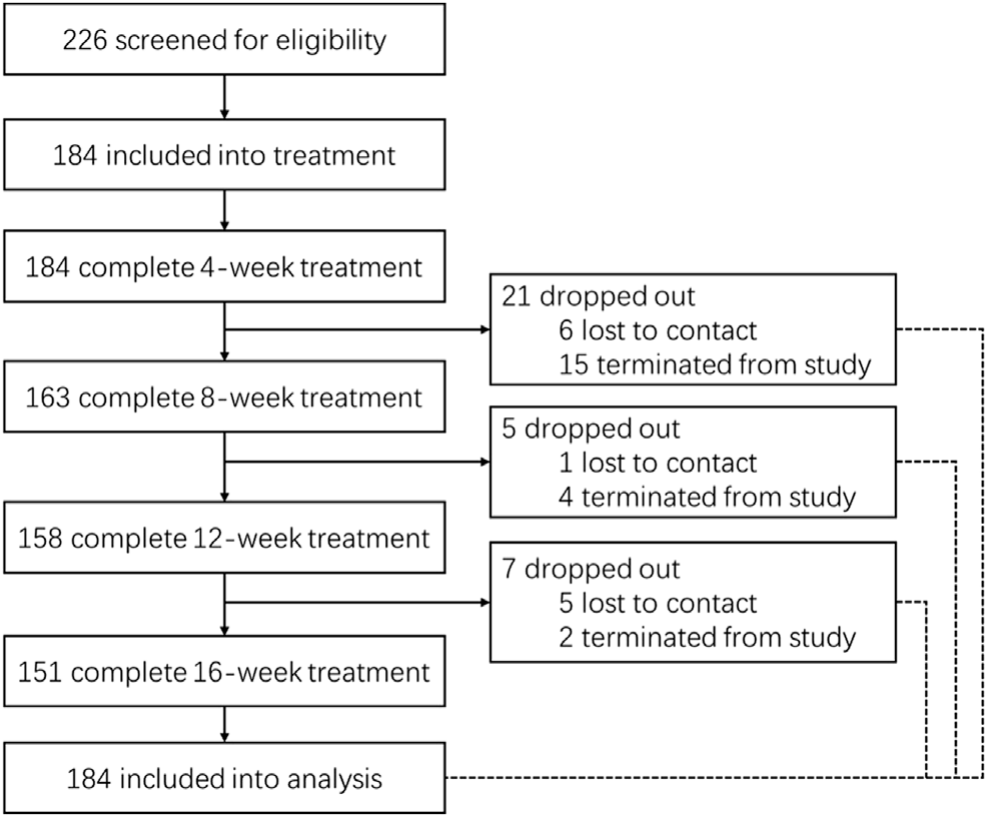
The flow chart of participants 12 participants were lost to contact while 21 participants were terminated from this study. This participants who showed syncope during the treatment with heart rate less than 30 bpm were terminated from this study and referred for further treatment, if appropriate.

Between January 2019 and January 2020, a total of 238 participants were screened for eligibility, of whom 184 were included in the study. After 16 weeks of treatment, 12 participants were lost to contact while 21 participants were terminated from this study and referred for permanent pacemaker implantation, with a total drop-out rate of 17.93% (Figure 3).

The baseline characteristics are shown in Table 1. The mean age of participants was 57.83 ± 17.73 years, 39.13% were male, 29.89% were current smokers, and the mean BMI was 19.88 ± 6.88 kg/m^2^. The average duration of symptomatic bradyarrhythmia was 7.67 ± 6.25 years; 29.89% had atrioventricular block while 37.50% had sinus node dysfunction. At baseline, 21.74% were receiving antihypertensive drugs, 17.39% receiving lipid-lowering drugs and 15.22% receiving aspirin. In addition, 35.33% experienced syncope in the month before treatment.

**TABLE 1:** Demographics of the study population.

Variables	Favorable outcome at 16 weeks (*n* = 112)	Unfavorable outcome at 16 weeks (*n* = 72)	Overall (*n* = 184)
Gender			
Men	40 (46.43%)	32 (44.44%)	72 (39.13%)
Women	72 (63.29%)	40 (55.56%)	112 (60.87%)
Age (years)			
Below 50	23 (20.54%)	12 (16.67%)	35 (19.02%)
50–59	31 (27.68%)	12 (16.67%)	43 (23.37%)
60–69	31 (27.68%)	21 (29.17%)	52 (28.80%)
70 and above	27 (24.11%)	27 (37.50%)	54 (29.35%)
Mean age (SD)	57.44±17.40	58.95±18.86	57.83±17.73
Education			
Primary school or less	18 (16.07%)	13 (18.06%)	31 (16.85%)
Middle school or high school	63 (56.25%)	41 (56.94%)	104 (56.52%)
College and higher	31 (27.68%)	18 (25.00%)	49 (26.63%)
Marriage status			
Living with spouse	95 (84.82%)	63 (87.50%)	158 (85.87%)
Living without spouse	17 (15.18%)	9 (12.50%)	26 (14.13%)
BMI			
<18.5 (underweight)	3 (2.68%)	1 (1.39%)	4 (2.17%)
18.5–24.9 (normal weight)	75 (66.96%)	48 (66.67%)	123 (66.85%)
≥25.0 (overweight and obesity)	34 (30.36%)	23 (31.94%)	57 (30.98%)
Mean BMI (SD)	19.91±6.87	19.80±6.98	19.88±6.88
Cigarette smoking			
Current smoker	27 (24.11%)	28 (38.89%)	55 (29.89%)
Never smoker or Ex-smoker	85 (75.89%)	44 (61.11%)	129 (70.11%)
Types of symptomatic bradyarrhythmia			
Atrioventricular block	24 (21.43%)	31 (43.06%)	55 (29.89%)
Sinus-node dysfunction	37 (33.04%)	32 (44.44%)	69 (37.50%)
Comorbidity			
Myocarditis	10 (8.93%)	8 (11.11%)	18 (9.78%)
Coronary artery disease	38 (33.93%)	34 (47.22%)	72 (39.13%)
Hypertension	41 (36.61%)	32 (44.44%)	73 (39.67%)
Diabetes mellitus	17 (15.18%)	14 (19.44%)	31 (16.85%)
Atrial fibrillation	11 (9.82%)	4 (5.56%)	15 (8.15%)
Atrial premature complexes	21 (18.75%)	13 (18.06%)	34 (18.48%)
Premature ventricular contractions	9 (8.04%)	8 (11.11%)	17 (9.24%)
Duration of symptomatic bradyarrhythmia			
1–5 years	50 (44.64%)	30 (41.67%)	80 (43.48%)
6–10 years	39 (34.82%)	28 (38.89%)	67 (36.41%)
10 years and above	23 (20.54%)	14 (19.44%)	37 (20.11%)
Mean duration (SD)	7.57±6.30	7.97±6.19	7.67±6.25
Use of medications			
Aspirin	22 (19.64%)	6 (8.33%)	28 (15.22%)
Lipid-lowering drug	22 (19.64%)	10 (13.89%)	32 (17.39%)
Hypoglycemic drug	8 (7.14%)	6 (8.33%)	14 (7.61%)
Anti-hypertensive drug	24 (21.43%)	16 (22.22%)	40 (21.74%)
ST-segment changes			
No	77 (76.24%)	53 (73.61%)	130 (70.65%)
Yes	35 (31.25%)	19 (26.39%)	54 (29.35%)
Syncope in the past month before treatment			
No	95 (84.82%)	24 (33.33%)	119 (64.67%)
Yes	17 (15.18%)	48 (66.67%)	65 (35.33%)

Regarding the safety of the Yuanjiang decoction, 12 participants (6.52%) reported xerostomia, 8 participants (4.35%) reported constipation, and 6 participants (3.26%) reported sleepiness. No participants left the study because of these adverse events.

Regarding compliance of the Yuanjiang decoction, a total of 41,216 bags were planned for use; among them, 37,064 bags (89.93%) were distributed to participants, and 30,665 bags (74.40%) were administered by the participants based on self-reports. Also, 142 participants (77.17%) completed at least 8-week Yuanjiang decoction.

**FIGURE 4:**
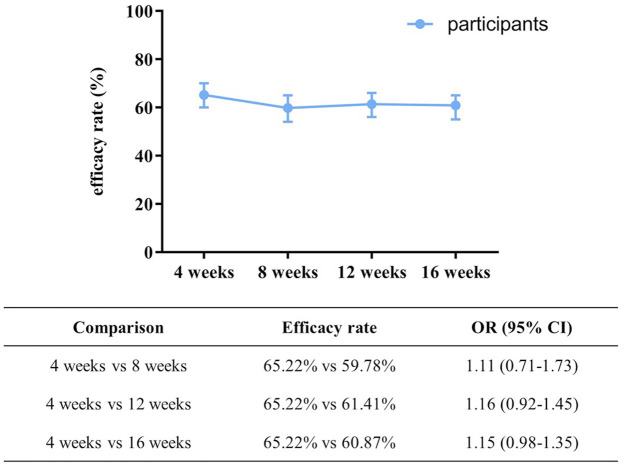
The proportion of participants with favorable treatment outcome at different time points. OR was adjusted for gender, age, education, marital status, BMI, cigarette smoking, types of symptomatic bradyarrhythmia, comorbidity, duration of symptomatic bradyarrhythmia, drug combination, ST-segment changes, completed at least 8-week treatment, and syncope in the past month before treatment.

For the primary outcome, as shown in Figure 4, based on ITT approach, the proportion of participants with favorable treatment outcome was 65.22% at 4 weeks, 59.78% at 8 weeks (OR: 1.11, 95% CI: 0.71–1.73), 61.41% at 12 weeks (OR: 1.16, 95% CI: 0.92–1.45) and 60.87% at 16 weeks (OR: 1.15, 95% CI: 0.98–1.35).

**FIGURE 5:**
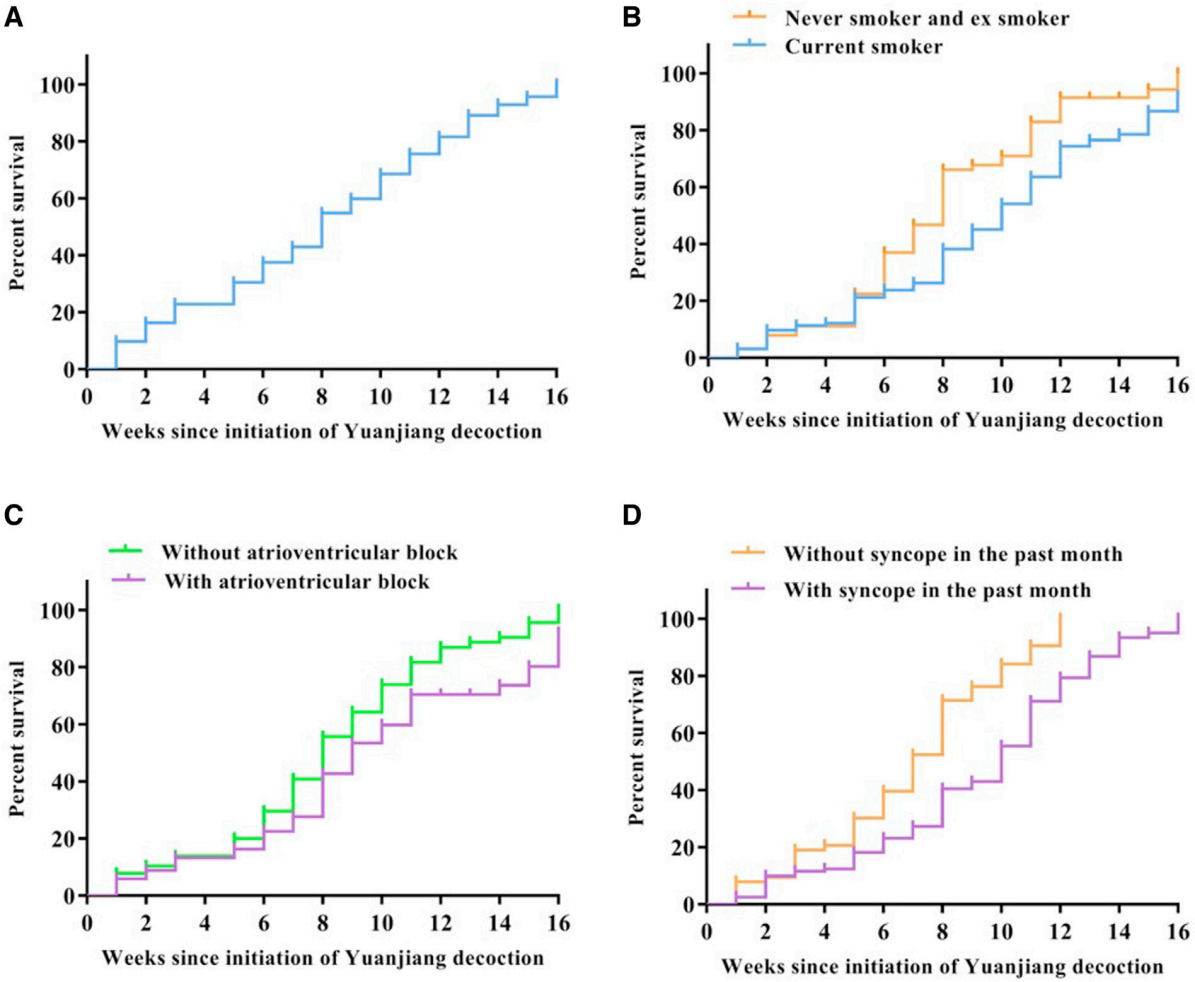
Cumulative incidence of clinical improvement. Clinical improvement is shown in the full cohort **(A)**, in the cohort stratified by smoking status [**(B)**, *p* = 0.013], in the cohort stratified by atrioventricular block [**(C)**, *p* = 0.017] and in the cohort stratified by syncope in the past month before treatment [**(D)**, *p* = 0.032].

Furthermore, the minimum heart rate was 34.85 ± 4.38 before treatment; this was significantly increased to 39.26 ± 5.32 at 4 weeks (*p* < 0.05), 39.73 ± 4.96 at 8 weeks (*p* < 0.05), 40.70 ± 5.01 at 12 weeks (*p* < 0.05) and 42.04 ± 4.87 at 16 weeks into treatment (*p* < 0.05). The results of the Kaplan-Meier analysis showed the median time to achieve a favorable treatment outcome was 8 weeks (Figure 5A). Clinical improvement was greater among ex-smokers or never smokers than smokers (Figure 5B), and among those without atrioventricular block than those with an atrioventricular block (Figure 5C), and among those who did not experience syncope in the past month before treatment than those who had syncope (Figure 5D).

After multifactor regression analysis (Table 2), the favorable treatment outcome at 16 weeks was significantly associated with the completion of at least 8 weeks treatment (OR: 2.053, 95% CI: 1.064–3.560), while unfavorable treatment outcome was significantly associated with an atrioventricular block (OR: 0.255, 95% CI: 0.083–0.784), current smoking (OR: 0.343, 95% CI: 0.027–0.487), and syncope in the month before treatment (OR: 0.321, 95%CI: 0.114–0.904).

**TABLE 2:** The Adjusted ORs for favorable treatment outcome at 16 weeks.

Variables	Model 1			Model 2	
	OR (95% CI)	*p*		OR (95% CI)	*p*
Gender					
Women	Ref			Ref	
Men	1.011 (0.487–3.100)	0.976		1.132 (0.339–3.773)	0.840
Age					
Below 50	Ref			Ref	
50–59	1.048 (0.340–3.230)	0.935		0.731 (0.132–4.046)	0.719
60–69	0.858 (0.288–2.552)	0.783		1.065 (0.178–6.386)	0.945
70 and above	0.685 (0.231–2.027)	0.494		1.063 (0.172–6.579)	0.948
Education					
Primary school or less	Ref			Ref	
Middle school or high school	0.667 (0.132–3.377)	0.624		0.349 (0.030–4.101)	0.403
College and higher	0.626 (0.117–3.359)	0.585		0.364 (0.035–4.485)	0.453
Marriage status					
Living without spouse	Ref			Ref	
Living with spouse	1.140 (0.407–3.192)	0.803		1.369 (0.277–6.765)	0.700
BMI					
<18.5 (underweight)	Ref			Ref	
18.5–24.9 (normal weight)	1.023 (0.097–3.354)	0.841		0.664 (0.505–8.882)	0.757
≥25.0 (overweight and obesity)	0.939 (0.088–2.003)	0.959		0.456 (0.031–6.792)	0.569
Cigarette smoking					
Never smoker or ex-smoker	Ref			Ref	
Current smoker	0.359 (0.026–0.512)	0.000		0.343 (0.027–0.487)	0.013
Types of symptomatic bradyarrhythmia					
Atrioventricular block	0.329 (0.119–0.915)	0.033		0.255 (0.083–0.784)	0.017
Sinus-node dysfunction	1.360 (0.612–3.022)	0.450		0.735 (0.331–1.634)	0.433
Comorbidity					
Myocarditis	0.619 (0.186–2.056)	0.434		0.978 (0.156–6.111)	0.981
Coronary artery disease	0.731 (0.294–1.817)	0.500		0.982 (0.279–3.460)	0.978
Hypertension	0.971 (0.370–2.552)	0.953		1.744 (0.418–7.282)	0.446
Diabetes mellitus	0.776 (0.263–2.292)	0.647		0.408 (0.084–1.975)	0.265
Atrial fibrillation	1.584 (0.397–6.320)	0.515		4.305 (0.384–8.319)	0.237
Atrial premature complexes	1.068 (0.367–3.105)	0.904		0.880 (0.170–4.559)	0.878
Premature ventricular contractions	0.673 (0.174–2.598)	0.565		0.354 (0.035–3.594)	0.380
Duration of symptomatic bradyarrhythmia					
1–5 years	Ref			Ref	
6–10 years	0.442 (0.164–1.91)	0.106		0.608 (0.183–2.021)	0.417
10 years and above	0.748 (0.272–2.052)	0.572		1.299 (0.362–4.668)	0.688
Use of medications					
Aspirin	3.502 (0.863–4.210)	0.079		3.488 (0.846–8.371)	0.084
Lipid-lowering drug	1.210 (0.371–3.939)	0.752		1.210 (0.372–3.942)	0.751
Hypoglycemic drug	0.753 (0.183–3.092)	0.694		0.749 (0.177–3.161)	0.694
Anti-hypertensive drug	0.532 (0.196–1.445)	0.216		0.533 (0.187–1.521)	0.240
ST–segment changes					
No	Ref			Ref	
Yes	0.909 (0.418–1.976)	0.810		0.964 (0.430–2.164)	0.930
Completed at least 8-week treatment					
No	Ref			Ref	
Yes	2.872 (0.835–3.014)	0.000		2.053 (1.064–3.560)	0.000
Syncope in the past month before treatment					
No	Ref			Ref	
Yes	0.303 (0.111–0.830)	0.020		0.321 (0.114–0.904)	0.032

## Discussion

To date, no therapy other than permanent pacemaker implantation has demonstrated sufficient efficacy for patients with symptomatic bradyarrhythmia. Our findings address several knowledge gaps about alternative treatment options for this condition. In our study, the favorable treatment outcome was observed in 60.87% of participants after 16 weeks of treatment, with completion of at least 8 weeks of treatment as an important contributing factor. Moreover, current smoking was significantly associated with unfavorable treatment outcome, suggesting that smoking cessation intervention should be implemented. However, the patients with atrioventricular block and those who experienced syncope before treatment showed unfavorable outcome, thus concomitant intensive supportive therapy should be provided for these patients.

The safety of Chinese herbal medicines is often queried. *Aristolochia fangchi* (Tankeu et al., 2016) and *Ephedra* (Odaguchi et al., 2019) are reported with a number of serious adverse effects on the cardiovascular and nervous system. The herbs of Yuanjiang decoction used in this study have been standard for decades. More importantly, although the participants in our study were elderly, had low SES and predominantly presented with severe symptoms, only 26 (14.13%) participants experienced mild adverse events, and no serious adverse effects were observed. However, it is important to also note that 21 of 184 participants were terminated from this trial due to worsening symptoms of bradyarrhythmia. Given the relatively short intervention period of our study, more evidence on the safety of Yuanjiang decoction is needed before its use becomes widespread.

The mechanism of Traditional Chinese medicine for symptomatic bradyarrhythmia is complex and remains unclear. Our previous study in rat models showed that Yuanjiang decoction could upregulate the expression of a hyper-polarization-activated cyclic nucleotide-gated cation channel (HCN4) and sodium channel protein type 5 subunit alpha (SCN5a) (Zhang et al., 2018). In addition, Dong and colleagues found that gingerol, the main component of *Zingiberis Rhizoma*, may promote the release of catecholamine from the adrenal medulla to antagonize the decrease of heart rate caused by the vagus nerve (Dong et al., 2018). Investigators also found that the tetrahydropalmatine in *Corydalis Rhizoma* could increase the activities of Na^+^-K^+^-ATPase and Ca^2+^-ATPase, promote Na^+^-Ca^2+^ exchange and increase intracellular Ca2+ concentration (Li et al., 2014). More studies are needed to clarify the mechanisms of Yuanjiang decoction.

Symptomatic bradyarrhythmia is difficult to treat and may have poor long-term outcomes without permanent pacemaker implantation. Since the first permanent pacemaker implantation in the late 1950s, clinical use of these devices has increased exponentially, and ongoing innovation in their design has resulted in a reduction in device-associated risks as well as a significant improvement in device performance and subsequent patient outcomes (Boriani et al., 2014). However, implantation of a permanent pacemaker is likely influenced by factors other than the patient’s age or associated medical conditions. Of note, the pacemakers are costly in China, resulting in a sizeable economic burden. Previous investigators have raised the concern that patients with low SES may have reduced access to more advanced technology (Muñoz et al., 2019). To address this unmet medical and social need, we developed the Yuanjiang decoction, with focus on clinical and cost-effectiveness at the same time. Within the analysis reported here, when clinical effects and economic costs are combined into a single metric, the use of Yuanjiang decoction is expected to yield substantial health benefits.

This study has important clinical implications. Traditional Chinese medicine and Western medicine have contrasting approaches to the prevention and treatment of diseases. Western medicine is superior in the rapid amelioration of symptoms, while Traditional Chinese medicine may provide a holistic approach for disease prevention and long-term care (Wong et al., 2001). With the findings of this study, we suggest that therapeutic strategies merging the merits of both TCM and Western medicine approaches could contribute to the improved management of patients with symptomatic bradyarrhythmia.

Despite the encouraging findings from this study, some aspects of the design may have limited the value of the data. Firstly, as explained above, there cannot be a control group for apparent ethical reasons in this compassionate-use study. To be able to assess the Yuanjiang decoction more meticulously, we are now in the process of collecting one-year follow-up data, which will be reported in the future. Secondly, because this was not a randomized controlled trial, the treatment benefits cannot be weighted properly, since it must be compared against historical controls. Also, although compatible with previous studies, especially in the Chinese population, the selection of primary outcome requires more supportive evidence. Fourthly, expectation bias caused by their belief in traditional Chinese medicine cannot be eliminated. Finally, owing to the nature of the program, several variables, such as cardiac electrophysiology, were not assessed (Schmidt et al., 2017).

## Conclusion

This compassionate-use study demonstrates that 3 out of 5 5 participants had encouraging outcomes after 16 weeks of treatment with Yuanjiang decoction, without serious adverse events. In addition, this study identified several key factors that affect the treatment outcome. These findings helped inform the design and assess the feasibility of a large-scale randomized clinical trial.

## Data Availability

The raw data supporting the conclusion of this article will be made available by the authors, without undue reservation.
