# Supplementary material for: Compassionate Use of Yuanjiang Decoction, a Traditional Chinese Medicinal Prescription, for Symptomatic Bradyarrhythmia
Source: Front Pharmacol. 2022 Apr 11;13:764930. doi: 10.3389/fphar.2022.764930 (PMC9035522; doi:10.3389/fphar.2022.764930)
Supplement: Supplementary file 1 [file DataSheet1.docx]

**Yuanjiang decoction for symptomatic bradyarrhythmia study**

**YJSBA Study Protocol**

YJSBA Study Steering Committee

Sponsored by Ministry of Science and Technology of China

Beijing, China

**1. INTRODUCTION**

Bradyarrhythmia is a severe cardiac arrhythmia in clinical practice, characterized by slow heart rate and hemodynamic disorders which can result in blood insufficiency. The reasons for bradyarrhythmia mainly included sinus-node dysfunction, atrioventricular-conduction disturbances, intraventricular block, etc ^[1]^. In severe cases, bradyarrhythmia could cause circulatory disturbance and endanger life.

The prevalence of bradyarrhythmia is increasing sharply owing to an ageing population ^[2]^. Despite its importance, few chemical medications such as atropine and isoproterenol could increase heart rate in a short time ^[3]^. More importantly, no approved medication is currently available for long-term use ^[4]^. In the absence of proven effective medication, clinically significant bradyarrhythmia with typical symptoms requires pacemaker implantation ^[5]^. The number of pacemaker implantation in China has greatly increased from 25,000 in 2007 to 73,000 in 2016 ^[6]^. However, the quality of life in patients with pacemaker implantation is severely affected due to high incidence of complications such as heart failure and atrial fibrillation ^[7]^, life-long follow-up demand ^[8]^ and its high cost ^[9]^. Therefore, many individuals refuse or cannot afford to pacemaker implantation, especially in low socioeconomic status (SES) population. This population is at the greatest risk for severe complications of BA, and there is an urgent need to find out an alternative option for them.

Traditional Chinese medicine has a long history of treating bradyarrhythmia, which may start from as early as 2,000 years ago ^[10]^. In recent years, a number of clinical trials showed TCM, such as Shenxian-Shengmai oral solution ^[11]^, Shensong Yangxin capsule ^[12]^, and Xinbao pill ^[13]^ has potential therapeutic effects for patients who had bradyarrhythmia without the need for pacemaker implantation. Considering the fact that o effective treatment was available for those who need pacemaker implantation but cannot afford it, it is of critical importance to supply alternative strategy for bradyarrhythmia.

**References**

1. Honarbakhsh S, Hunter L, Chow A, Hunter RJ. Bradyarrhythmias and pacemakers. BMJ, 2018, 360: k642.
2. Ma LY, Chen WW, Gao RL, et al. China cardiovascular diseases report 2018: an updated summary. J Geriatr Cardiol, 2020, 17 (1): 1-8.
3. Neumar RW, Otto CW, Link MS, et al. Part 8: adult advanced cardiovascular life support: 2010 American Heart Association Guidelines for Cardiopulmonary Resuscitation and Emergency Cardiovascular Care. Circulation, 2010, 122 (18 Suppl 3): S729-S767.
4. Epstein AE, DiMarco JP, Ellenbogen KA, et al. 2012 ACCF/AHA/HRS focused update incorporated into the ACCF/AHA/HRS 2008 guidelines for device-based therapy of cardiac rhythm abnormalities: a report of the American College of Cardiology Foundation/American Heart Association Task Force on Practice Guidelines and the Heart Rhythm Society. J Am Coll Cardiol, 2013, 61 (3): e6-e75.
5. Hua W, Zhang S. Forge ahead and learn from the brilliant-ten years review of Chinese cardiac pacing career (In Chinese). Chinese Journal of Cardiac Arrhythmias, 2018, 22 (01): 8-10.
6. Gillam MH, Pratt NL, Inacio M, Shakib S, Sanders P, Lau DH, Roughead EE. Rehospitalizations for complications and mortality following pacemaker implantation: A retrospective cohort study in an older population. Clin Cardiol, 2018, 41 (11): 1480-1486.
7. Johansen JB, Jørgensen OD, Møller M, Arnsbo P, Mortensen PT, Nielsen JC. Infection after pacemaker implantation: infection rates and risk factors associated with infection in a population-based cohort study of 46299 consecutive patients. Eur. Heart J, 2011, 32 (8): 991-998.
8. Mond HG, Proclemer A. The 11th world survey of cardiac pacing and implantable cardioverter-defibrillators: calendar year 2009: a World Society of Arrhythmia's project. Pacing Clin Electrophysiol, 2011, 34 (8): 1013-1027.
9. Clementy N, Carion PL, de Leotoing L, Lamarsalle L, Wilquin-Bequet F, Brown B, Verhees KJP, Fernandes J, Deharo J. Infections and associated costs following cardiovascular implantable electronic device implantations: a nationwide cohort study. 2018, 20 (12): 1974-1980.
10. Clémenty N, Fernandes J, Carion PL, de Léotoing L, Lamarsalle L, Wilquin-Bequet F, Wolff C, Verhees K, Nicolle E, Deharo JC. Pacemaker complications and costs: a nationwide economic study. J Med Econ, 2019, 22 (11): 1171-1178.
11. Chan K. Chinese medicinal materials and their interface with Western medical concepts. J Ethnopharmacol, 2005, 96 (1-2): 1-18.
12. Gao J, Wang T, Yao X, Xie W, Shi X, He S, Zhao T, Wang C, Zhu Y. Clinical evidence-guided network pharmacology analysis reveals a critical contribution of β1-adrenoreceptor upregulation to bradycardia alleviation by Shenxian-Shengmai. BMC Complement Altern Med, 2019, 19 (1): 357.
13. Liu Y, Li N, Jia Z, Lu F, Pu J. Chinese medicine shensongyangxin is effective for patients with bradycardia: results of a randomized, double-blind, placebo-controlled multicenter trial. Evid Based Complement Alternat Med, 2014, 2014: 605714.

**2. AIMS**

To explore the safety and efficacy of Yuanjiang decoction, a traditional Chinese medicinal prescription, for symptomatic bradycardiasymptomatic bradyarrhythmia on a compassionate-use basis.

**3. STUDY PARTICIPANTS**

**3.1 SELECTION OF STUDY PARTICIPANTS**

Only permanent residents of nationality of the People's Republic of China were eligible for inclusion. The trial adhered to the Declaration of Helsinki.

**3.2 ETHIC REVIEW**

This study was reviewed and approved by the institutional review board of Xiyuan Hospital of China Academy of Chinese Medical Sciences before the start of the study (No. 2018XLA035-2) and registered in Chinese Clinical Trial Registry (No. ChiCTR-1800018464).

All participants signed informed consent and received financial compensation for trial participation time and travel expenses.

As this was a compassionate-use study, the Xiyuan Hospital of China Academy of Chinese Medical Sciences established a specific Data and Safety Monitoring Board (DSMB) to monitor the study once per month and received summaries of study conduct, safety, and efficacy.

**3.3 STUDY SITES**

Xiyuan Hospital, China Academy of Chinese Medical Sciences, Beijing, China.

**3.4 INCLUSION CRITERIA**

(1) aged 18 to 90 years;

(2) documented symptomatic bradyarrhythmia;

(3) Class I indications of permanent pacemaker implantation;

(4) low SES and cannot afford to pacemaker implantation due to economy reasons;

(5) voluntarily participated in this trial and signed the informed consent. In addition, women were required to have a negative urine pregnancy test before treatment.

**3.5 EXCLUSION CRITERIA**

(1) pregnant and lactating;

(2) allergic predisposition and drug allergy;

(3) complicated with severe liver disease, severe renal dysfunction, persistent atrial fibrillation, acute myocardial infarction, stroke, hematopoietic system abnormalities and mental illness in the past 3 months;

(4) drug-induced transient secondary bradycardia;

(5) athletes with low heart rate but normal sinoatrial node function, without bradycardia-related symptom;

(6) participated in other clinical trials in the past month;

(7) taking any Chinese medicinal herbs in the past month.

**4. YUANJIANG DECOCTION PREPARATION**

All participants received 16 weeks of Yuanjiang decoction, self-administered, 200 mL orally, twice per day and supportive therapy was provided at the discretion of the clinicians.

The Yuanjiang decoction was composed of 6 herbs: Alpiniae Officinarum Rhizoma, 12g; Zingiberis Rhizoma, 6g; Cinnamomi Cortex, 6g; Poperis Longi Fructus,12g; Corydalis Rhizoma, 15g; Curcumae Radix, 12g (Figure 1).


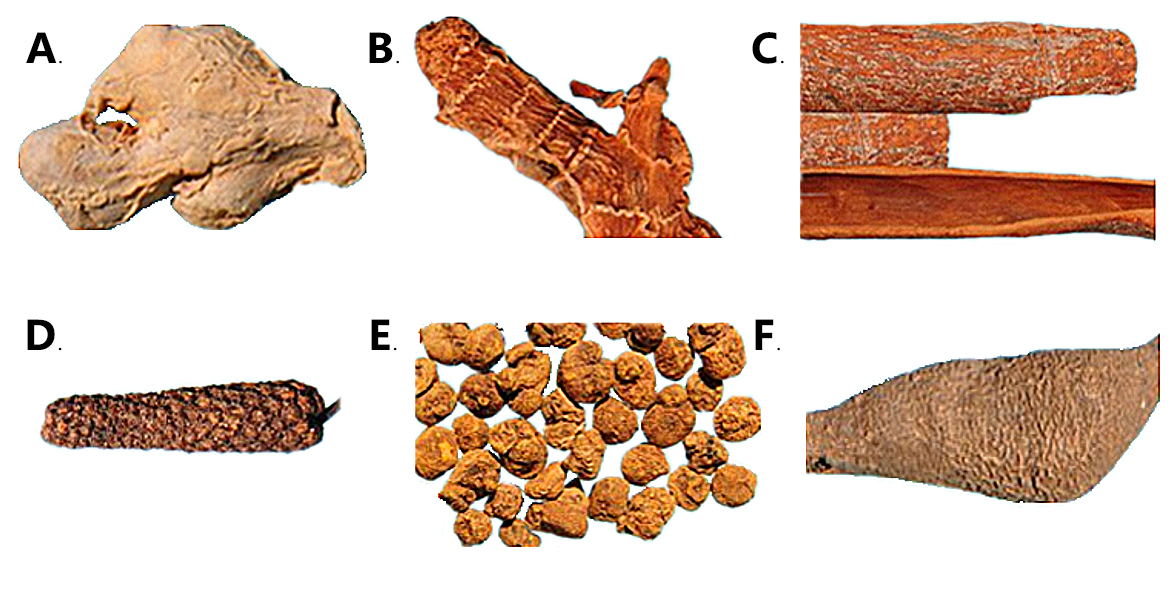


**Figure 1. Example of 6 herbs of Yuanjiang decoction**

Note: A, Zingiberis Rhizoma; B, Alpiniae Officinarum Rhizoma; C, Cinnamomi Cortex; D, Poperis Longi Fructus; E, Corydalis Rhizoma; F, Curcumae Radix.

The procedure for preparing decoction is shown in Figure 2. In brief, a specialist pharmacist was responsible for decoction management and quality control. Before decoction preparation, all herbs were tested for heavy metals, microbial contamination, and residual pesticides. All the herbs were then weighed and transferred into an automatic decocting machine. Here, they were immersed in water for 30 minutes and decocted under 110 degree (Celsius) and 150 kPa for 30 minutes. Finally, the decoctions were automatically packed with a specification of 200 mL. The criteria for the quality of the herbs used were in accordance with the 2015 Chinese pharmacopoeia.


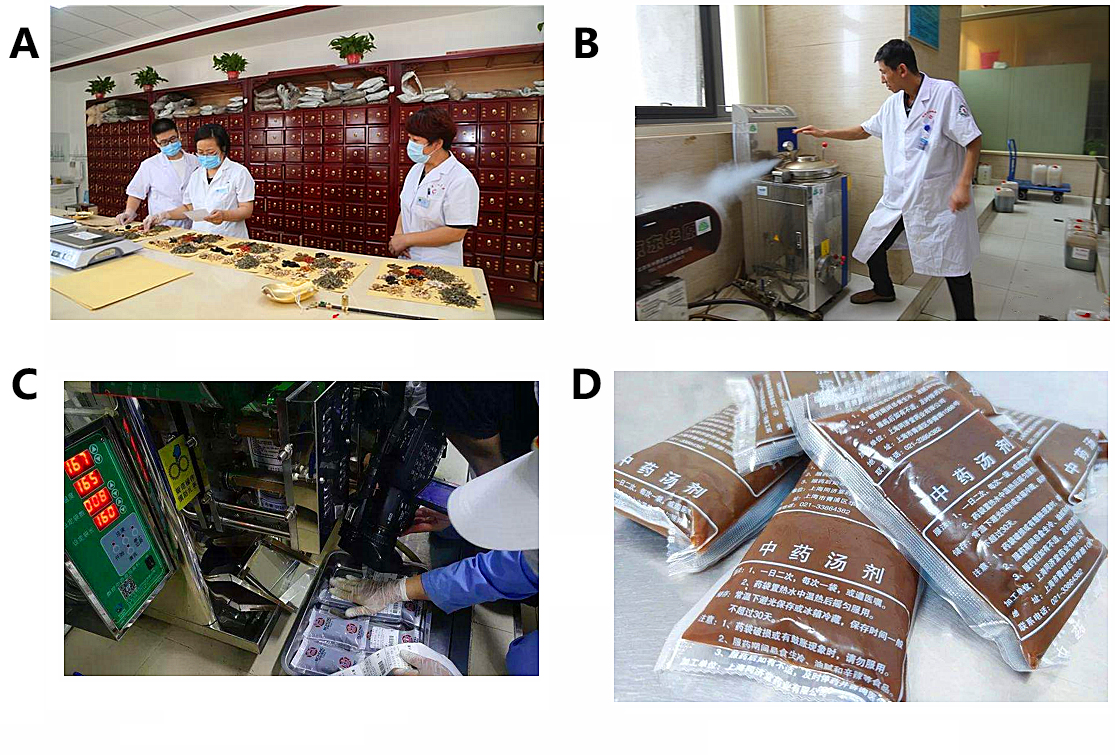


**Figure 2. The procedure for preparing decoction**

Note: A, herbs preparation; B, machine decocting; C, decoction packing; D, decoction packages distributed to participants.

Participants were instructed to drink the Yuanjiang decoctions 30 min after breakfast and dinner, respectively, and not to take any other drugs, tea or coffee within half an hour before and after taking the decoction to avoid possible reactions with the Chinese medicine ingredients.

**5. IMPLEMENTATION PROCESS**

**5.1 SCREENING PERIOD**

- Sign informed consent
- Check for Inclusion and exclusion criteria
- Medical History, demographic information and medication
- Holter monitoring
- Enrollment

**5.2 ADMINISTRATION PERIOD（0 weeks to 4 weeks）**

- Administration of Yuanjiang decoction
- Record adverse event if happen
- Holter monitoring in 4 weeks

**5.3 VISIT 1（4 weeks±2 days）**

- Analysis of the primary outcome by DSMB to decision about whether or not to continue administration.

**5.4 ADMINISTRATION PERIOD（4 weeks to 8 weeks）**

- Administration of Yuanjiang decoction every day
- Record adverse event if happen
- Holter monitoring in 8 weeks

**5.5 VISIT 2（8 weeks±2 days）**

- Analysis of the primary outcome by DSMB to decision about whether or not to continue administration.

**5.6 ADMINISTRATION PERIOD（8 weeks to 12 weeks）**

- Administration of Yuanjiang decoction every day
- Record adverse event if happen
- Holter monitoring in 12 weeks

**5.7 VISIT 3（12 weeks±2** **days）**

- Analysis of the primary outcome by DSMB to decision about whether or not to continue administration.

**5.8 ADMINISTRATION PERIOD（12 weeks to 16 weeks）**

- Administration of Yuanjiang decoction every day
- Record adverse event if happen
- Holter monitoring in 12 weeks

**5.9 Visit 4（16 weeks ±2）**

- Analysis of the primary outcome by DSMB

**6. OUTCOME**

**6.1 Demographic Information**

**6.2 Medication**

**6.3 Medical History**

- The definition of Class I indications of permanent pacemaker implantation was based on the ACC/AHA/HRS 2008 Guidelines.
- The definitions of sinus node dysfunction, sinus pause, sinoatrial exit block and atrioventricular block were based on 2018 ACC/AHA/HRS Guideline.
- The low SES was defined as monthly income less than 1600 China Yuan (CNY), approximately 230 US dollars.
- The definition of myocarditis was based on the Current State of Knowledge on Aetiology, Diagnosis, Management, and Therapy of Myocarditis.
- The definition of coronary artery disease was based on the 2016 ACC/AHA Guideline.
- The definition of hypertension was based on the 2018 ESC/ESH Guideline. The definition of Paroxysmal AF and atrial premature complexes was based on the 2015 ACC/AHA/HRS Guideline.
- The definition of premature ventricular contractions was based on the 2017 AHA/ACC/HRS Guideline.
- The definition of diabetes mellitus was based on the Update on Prevention of Cardiovascular Disease in Adults with Type 2 Diabetes Mellitus in Light of Recent Evidence
- The definition of current smoking was having smoked 100 cigarettes in one’s lifetime and currently smoking cigarettes.

**6.4 The primary outcome was defined as bradycardia-related symptom improvement, along with either or both of minimum heart rate increased more than 5 bpm, and minimum heart rate more than 40 bpm)**

- The bradycardia-related symptom was assessed by using a self-completed visual analogue scales (VAS). The stem question for the symptom specific VAS was, “How severe was your [X] today?” in which “X” was replaced with the patient’s specific symptom. For VAS questions, the patient responded by making a mark between 0 mm (no symptoms) and 100 mm (maximum intensity for the symptom in question) on a horizontal line.

**6.5 Adverse event**

**7. DATA MANAGEMENT**

**7.1 ELECTRONC DATA MANAGEMENT SYSTEM**

All data collected from each participate were conveyed through electronic data capture (EDC) to the general center server at the Institute of clinical pharmacology, Xiyuan hospital.

**7.2 DATA SECURITY**

Confidentiality agreement was established and signed by all participating organizations to ensure behavioral specification for all personnel. In addition, secrecy system was constructed to prevent data leaks, and the data bases at various ranks differ in permission.

**8. COMMON STATISTICAL ANALYTICAL METHODS**

Intent-to-treat approach was applied in this trial, and the participants who were lost to contact or terminated from the study were considered to be unfavorable treatment outcome. The measurement data were represented with means±SDs. The t test was used for comparison which met Gaussian distribution and homogeneity of variance, whereas nonparametric test was used for comparison which did not met homogeneity of variance. The chi-square test was used for numeration data. The logistic regression analysis was used to calculate the relationship between possible influence factors and efficacy, represented with OR value and 95% confidence interval. The Kaplan-Meier curve was used for analyses of optimal treatment time.

Missing data on the primary outcome were imputed using the multiple imputation method under the missing-at-random assumption. SPSS 19.0 statistical software (SPSS, Inc) was used for statistical analysis. P<0.05 was taken as statistical significance.

The authors had no access to information that could identify individual participants during or after data collection

**The Informed Consent (in Chinese)**

元姜方治疗缓慢性心律失常的双向队列研究试验

知情同意书.

知情告知页

尊敬的患者：您好！

您的医生已诊断您患有缓慢性心律失常疾病。欢迎您自愿参加**元姜方治疗缓慢性心律失常的队列研究临床试验**，并对您的参加表示衷心的感谢。在决定是否参加之前，您有必要了解本试验的目的、试验药品、可能给您带来的风险、试验中期望您所做的事以及您作为受试者的权利。请您仔细阅读这份受试者须知。

**1、研究背景**

缓慢性心律失常( bradycardia) 是以心率减慢为特征的疾病，美国心脏病学会( American Heart Association，AHA) 规定成人心率低于60次/min即为缓慢性心律失常，包括窦房结自律性和窦房传导紊乱。临床常见的类型为病态窦房结综合征、房室传导阻滞等，主要表现为乏力、症状性低血压、心悸、胸闷及脑供血不足所致的头晕、头痛、黑矇等。尽管植入起搏器可有效提高患者心率，缓解症状，但起搏器治疗花费昂贵，且需要终生随访治疗，很大程度影响了患者的生活质量。缓慢性心律失常可以出现在各个年龄段，但老年人群中的发病率最高，平均发病年龄为68岁。有研究显示在年龄大于65岁的心脏病患者中病窦的发病率为0.17%。因此，对于缓慢性心律失常，有效的药物治疗显得十分重要。中医药认为病态窦房结综合征的起搏功能减退乃是中医心之阳气虚衰的病理表现。心阳虚衰则不能鼓舞血脉、温养脏腑，表现出心悸、胸闷、畏寒肢冷、等一系列症状，故临床认为心肾阳衰，阴寒内聚，心脏气血两虚，是形成本病的根本原因。

翁维良从事活血化瘀研究40年余，提出“百病多瘀”，“心病多瘀”理论，总结出临床治疗心血管疑难杂症的活血化瘀十二法，针对起搏功能异常所造成的缓慢性心律失常，善用活血化瘀，在郭士魁芳香温通法的基础上，针对现代社会疾病谱的特点，归纳总结出治疗病窦、房室传导阻滞等缓慢性心律失常病证的温阳活血法，临床疗效显著。在明确疗效特点的基础上，整理分析诊疗方案中药味-剂量-疗效的证效/量效关系，挖掘处温阳活血法治疗缓慢性心律失常的核心处方。处方最终确定为：肉桂、干姜、高良姜、荜茇、延胡索、郁金等。

**2、研究介绍**

本试验已获得中国中医科学院西苑医院伦理委员会批准。研究目的为探讨元姜方（核心方）治疗缓慢性心律失常的临床疗效。本研究为元姜方治疗缓慢性心律失常的**前瞻性队列研究，纳入2018年6月1日至2019年1月1日，就诊于翁维良特需门诊的缓慢性心律失常患者的临床诊疗资料**。研究过程严格按照《赫尔辛基宣言》原则进行。该研究在中国中医科学院西苑医院特需门诊，对缓慢性心律失常进行中药元姜方（核心方）治疗，对其治疗效果进行分析。

**3、哪些人适宜参加研究**

符合病态窦房结综合征/房室传导阻滞诊断标准。

**4、哪些人不宜参加研究**

①经检查证实为IV级冠心病心绞痛患者或急性心肌梗塞、心功能IV级者。

②合并重度高血压患者、明显血流动力学障碍者。

③合并肝功能不全、肾功能不全，造血系统异常等严重原发性疾病及神经病患者。

④妊娠或哺乳期妇女。

**⑤抗心律失常药物致缓慢性心律失常（服用羟氯喹导致房室传导阻滞，地尔硫卓、普罗帕酮、美西律、胺碘酮、β受体阻滞剂等引起的缓慢性心律失常，**

1. **总体过程**

⑴入选研究前，患者应在开服服用药物一周内进行动态心电图检测。

⑵研究方法：本试验为观察性研究，患者在翁老门诊接受治疗期间，对您的诊疗数据进行收集及分析总结

⑶治疗后观察时点：治疗后复查心电图

⑷要您配合的其他事项:如复诊日期、复诊需要配合的检查等。

流程如下

筛选入组期

- 签署知情同意书
- 入选、排除标准核查
- 问诊（取得人口学信息、既往病史、用药史、等）
- 提供近一月内24小时动态心电图检查
- 入组

受试者给药期

- 给药
- 记录伴随用药及不良事件

访视2：出组

- 24小时动态心电图检查
- 记录伴随用药及不良事件
- 出组

请您按医生和您约定的随访时间来就诊。您的随访非常重要，因为医生将判断您接受的治疗中否真正起作用。

请您按医生指导用药，并请您在每次用药后及时、客观地记录。同时要记录试验期间您由于其它疾病必须继续服用的药物。

在试验期间除方案中规定的用药外，请不要使用其它治疗缓慢性心律失常的中西药，及与本病治疗相关的其它药物。

**试验期间请忌食辛辣油腻物品。**若您需要其它治疗，请事先与您的医生联系。

1. **您的权利和利益**

（1）研究人员将向您介绍试验药品及试验安排，参加与否完全遵循自愿的原则。研究者向您报告所有与您有关的事件，以便您随时决定是否继续参加。如果您有任何问题，您可打电话或直接向研究者咨询。您将在研究期间获得良好的医疗服务。您可以拒绝参加此项研究，或在研究过程中的任何时间退出研究，这都不会影响您和医生的关系，都不会影响对您的医疗或有其他方面利益的损失, 您可以随时退出本研究，而不会受到歧视或不公正待遇，您的医疗待遇与权益不会受到影响。您不必为了治疗您的疾病而必须选择参加本项研究。如果由于药物的原因使您退出研究时，您若将病情变化告诉医生，完成相应的体格检查和理化检查，这将对您本人的健康和整个研究都十分有利。

（2）您和社会将可能从本项试验中受益。此种受益包括您的病情可能获得改善，以及您将获得一次使用一种治疗缓慢性心律失常新药的机会。同时您的参与也将为研制一种治疗本病的新药做出贡献。本项研究可能帮助开发出一种新治疗方法，以用于患有相似病情的其他病人。

**7、您的义务**

坚持自愿参加的原则，在试验开始之前签署知情同意书。

遵循临床试验方案，听从研究者的统一安排。配合研究者完成试验任务。

**8、可能出现的不良反应和安全措施**

迄今为止，在已有的文献数据库中检索后暂未发现本品临床应用过程中发生不良反应。且本研究为观察性研究，仅记录正常诊疗过程中的相关医疗信息，进行整理分析。

如果在研究中您出现任何不适，或任何意外情况，不管是否与药物有关，均应及时通知您的医生，他/她将对此作出判断和医疗处理。

医生和研究者将尽全力预防由于本研究可能带来的伤害。如果在临床研究中出现不良事件，医生将尽力救治，医学专家将会鉴定其是否与研究药物有关。确定属实的翁维良名老中医工作站对与研究相关的损害提供治疗的费用及相应的赔偿。这一点已经在我国《药物临床试验质量管理规范》中做出了规定。

**7、终止您参加试验的理由**

⑴、研究者判定进行试验对您有害。

⑵、如果您出现了研究者发现疗效太差等情况（中止标准），

⑶、您未按医生的指导用药。

⑷、研究过程中发生了严重不良反应。

发生了上述情况，研究者有权不征得您的同意而终止您参加试验。

1. **保密性**

一切有关于你的信息，包括您的身份、医疗史、病情、体检以及实验室检查结果等，都将在法律允许的范围内得到严格的保密。只有授权的研究者、伦理委员会及研究立项部门才能查阅您的记录，食品药品监督管理部门被允许查阅您的与本研究相关的医疗记录，以证实本研究所收集资料的真实性和准确性，但不涉及您的个人详细资料。您的姓名不会出现在任何与此项研究相关的公开资料或报告中。

1. **发表**

无论研究结果如何，我们都将尽力将研究结果发表。

**感谢您阅读以上资料。如果您决定参加临床研究，请告诉您的医生，他/她会为您安排一切有关临床研究的事宜。**

**请您保留这份资料。**

知情同意书签字页

试验名称:

申办者：

国家食品药品监督管理局临床研究批件号：

同意声明

我已经阅读了上述有关本研究的介绍，而且有机会就此项研究与医生讨论并提出问题。我提出的所有问题都得到了满意的答复。

我知道参加本研究可能产生的风险和受益。我知晓参加研究是自愿的，我确认已有充足时间对此进行考虑，而且明白：

- 我可以随时向医生咨询更多的信息。
- 我可以随时退出本研究，而不会受到歧视或不公正待遇，医疗待遇与权益不会受到影响。

我同样清楚，如果我中途退出研究，特别是由于药物的原因使我退出研究时，我若将病情变化告诉医生，完成相应的体格检查和理化检查，这将对我本人和整个研究十分有利。

如果因病情变化我需要采取任何其他的药物治疗，我会在事先征求医生的意见，或在事后如实告诉医生。

我将获得一份经过签名并注明日期的知情同意书副本。同时我允许国家食品药品监督管理局/国家相关部门、伦理委员会及相关研究人员审阅记录。

最后，我决定同意参加本项研究。

患者签名： 身份证号□□□□□□□□□□□□□□□□□□

患者联系电话： 手机号： ＿＿＿＿年＿＿月＿＿日

法定代理人签名： 身份证号□□□□□□□□□□□□□□□□□□

联系电话： 手机号： ＿＿＿＿年＿＿月＿＿日

————————————————————————————————————————

我确认已向患者解释了本试验的详细情况，包括其权利以及可能的受益和风险，并给其一份签署过的知情同意书副本。

医生签名： 日期：＿ ＿ ＿ ＿ 年 ＿ ＿ 月 ＿ ＿ 日

医生的工作电话： 手机号：

西苑医院医学伦理委员会办公室联系电话：010 62835646

**The Case Report Form (in Chinese)**

| 编号  □□□□ | 受试者姓名拼音缩写 | 研究类型 |
| --- | --- | --- |
|  |  | 队列研究 |

**元姜方治疗缓慢性心律失常的双向队列研究**

**研究病历**

患者姓名（拼音缩写）：

医院名称：

研究者姓名：

入组日期：

**负责单位：中国中医科学院西苑医院**

**研究病历填写说明**

1、合格者填写正式观察表。

2、表中凡有“□”的项，请在符合的条目上划“√” 。

3、所有检查项目均须填写，因故未查或漏查，请填写“ND” ；具体用药剂量和时间不明，请填写“NK” 。

4、观察表的每页均须填写试验中心号、编号、患者姓名拼音缩写、就诊日期，观察医生必须签署姓名和日期。

5、所有表格的日期都以年/月/日的形式表示。

6、表格填写务必准确、清晰，任何数据禁止擦除或涂墨，如有错误发生，可在错误处上方书写正确值，将错误值划上“--”，修改者签名并加注日期，必要时说明理由。

7、试验期间应如实填写合并用药记录表。

8、各实验室检查的检查报告、化验单，均应贴在表格的最后一面。

9、本表格用钢笔或签字笔填写，不得用铅笔或圆珠笔填写。

10、姓名缩写：患者姓名拼音缩写四格需填满，两字姓名填写两字拼音前两个字母；三字姓名填写三字首字母及第三字第二字母；四字姓名填写每一个字首字母。举例：王红 WAHO，王艳华 WYHU，欧阳松下 OYSX。

**观察指标流程图**

| **流程** | **入选前** | **治疗期** | | | | |
| --- | --- | --- | --- | --- | --- | --- |
| **观察时间** | **0** | **1个月** | **3个月** | **6个月** | **9个月** | **1年** |
| **观察指标** |  |  |  |  |  |  |
| **一般临床资料** | **√** |  |  |  |  |  |
| 动态心电图 | **√** | **√** | **√** | **√** | **√** | **√** |
| 药物相互作用 | **√** | **√** | **√** | **√** | **√** | **√** |
| 不良事件 |  | **√** | **√** | **√** | **√** | **√** |
| **依从性观察** |  | **√** | **√** | **√** | **√** | **√** |
| **记录合并用药** | **√** | **√** | **√** | **√** | **√** | **√** |

注：终点指标包括①主要终点指标：心源性死亡、起搏器植入术；②次要终点指标：全因死亡、因ACS/心衰/恶性心律失常入院治疗、脑卒中及其他新发血管性疾病

**病例入选与排除标准**

| **入选标准：** | **是**_1_ | **否**_0_ |
| --- | --- | --- |
| 符合心动过缓诊断标准者； | 🞎 | 🞎 |
| **排除标准：** |  |  |
| 1. 经检查证实为IV级冠心病心绞痛患者或急性心肌梗塞、心功能IV级者。 | 🞎 | 🞎 |
| 2. 合并重度高血压患者、明显血流动力学障碍者。 | 🞎 | 🞎 |
| 3. 高血压控制不良(收缩压≥160mmHg且舒张压≥100mmHg)； | 🞎 | 🞎 |
| 4.合并肝功能不全、肾功能不全，造血系统异常等严重原发性疾病及神经病患者。 | 🞎 | 🞎 |
| 5. 妊娠或哺乳期妇女。 | 🞎 | 🞎 |
| 如果以上任何一项回答“是”，则受试者不能进入研究。 | | |

**访视1导入期**

**____年____月____日**

**人口统计学**

| 姓名 |
| --- |
| 性别 □男 □女 |
| 年龄 |
| 民族 |
| 工作性质：□脑力劳动 □体力劳动 □离退 □其他 |
| 身高______cm 体重_____kg BMI_____ |

**患者资料记录**

| 家族史 | □心动过缓 □冠心病 □高血压 □糖尿病 □其他 □不详 |
| --- | --- |
| 吸烟史 | □无 □有：______年  是否已戒烟：□否； □是 |
| 饮酒史 | □无 □有：______年  是否已戒酒：□否； □是 |
| 高血压病史 | □无 |
|  | □有：病程_____年；最高血压___/___mmHg  是否服用药物治疗：□无 □有：___种（药物名称_______）  血压是否稳定： □是 □否 |
| 糖尿病病史 | □无 |
|  | □有：病程_____年 血糖是否稳定： □是 □否  是否服用药物治疗：□否  □有：□胰岛素 □口服药物 |
| 冠心病病史 | □无 |
|  | □有：病程_____年 |
| 心肌炎病史 | □无 □有 □不详 |
| 心房颤动病史 | □无 |
|  | □有 病程_____年 □阵发性 □永久性  是否服用药物治疗：□否 □是（药物名称： ）  是否射频消融：□否 □是 |
| 其他疾病 |  |
| 药物过敏史 | □0 无； □1 有 请记录_________ |

**CHA2DS2评分**

| Congestive Heart Failure充血性心力衰竭 | □否（0分） □是（1分） |
| --- | --- |
| Hypertension 高血压 | □否（0分） □是（1分） |
| Age 年龄≥75岁 | □否（0分） □是（1分） |
| Diabetes Mellitus 糖尿病 | □否（0分） □是（1分） |
| Previous Stoke/TIA/syncope 晕厥 | □否（0分） □是（2分） |
| 总分（6分） |  |

**CHA2DS2-VASc评分**

| Congestive Heart Failure充血性心力衰竭 | □否（0分） □是（1分） |
| --- | --- |
| Hypertension 高血压 | □否（0分） □是（1分） |
| Vascular Disease 血管性疾病 | □否（0分） □是（1分） |
| Female 女性 | □否（0分） □是（1分） |
| Age 年龄65-74岁 | □否（0分） □是（2分） |
| Diabetes Mellitus 糖尿病 | □否（0分） □是（1分） |
| Previous Stoke/TIA/syncope 晕厥 | □否（0分） □是（2分） |
| 总分（9分） |  |

**心动过缓病史及治疗史**

| 心动过缓病程（合计） | | | _________月 |
| --- | --- | --- | --- |
| 是否接受药物治疗 | | | □否 □是（药物名称：） |
| 是否植入起搏器 | | | □否 □是（手术日期：） |
| Holter  检查记录 | □无  □有 _____次 | | |
|  | 心率： | 最慢心率_____，最快心率______，平均心率______  总心搏数_____，最长R-R间期_____s  停搏＞2s：____s，  心动过速（＞130bpm）有____跳，占总心率_____%  心动过缓（＜45bpm）有____跳，占总心率_____% | |
|  | 心率  变异 | SDNN_____ SDNN Idx_____ PNN50_____  rMSSD _____ SDANN ______ 三角指数 | |
|  | 室上性节律 | 总数____次，平均（小时）：  单发： 成对：  二联律： 三联律：  室上速：  最长室上速次数：  最大心率室上速 | |
|  | 室性  节律 | 总数____次，平均（小时）：  单发： 成对：  二联律： 三联律：  室速总数：_____阵 RonT：_____阵  最快心率的室速：  最小心率的室速 | |
|  | 房颤  分析 | 房颤心搏：  持续时间： 发生次数  ＞1.5s心搏有：____次  ＞2s心搏有：____次 | |
|  | 结论 |  | |
| 阿托品  试验 | □无  □有 结论： | | |

**中医主症：**

| 中医主症 | 记分 | 症状分级记分标准 |
| --- | --- | --- |
| 心 悸 |  | 0分：无。3分：偶发心悸，可自行缓解；6分：频繁发作，但能坚持工作。9分：心悸持续不解，影响生活和工作。 |
| 胸 闷 |  | 0分：无。3分：偶感胸闷，可自行缓解。6分：胸闷发作较频繁，但不影响正常生活和工作。9分：胸闷持续不解，影响生活和工作。 |
| 气 短 |  | 0分：无。2分：活动后气短。4分：稍动即气短。6分：平时亦感气短。 |
| 乏 力 |  | 0分：无。2分：重度活动感乏力；4分：中度活动感乏力。6分：轻度活动即乏力。 |
| 头 晕 |  | 0分：无。2分：偶感头晕，可自行缓解；4分：头晕发作较频繁，但不影响正常生活和工作。6分：头晕持续不解，影响生活和工作。 |
| 总分 |  | |

**中医兼症：**

|  | |  |  手足心热 | □1轻 □2中 □3重 |
| --- | --- | --- | --- | --- |
|  盗汗 | | □1轻 □2中 □3重 |  自汗 | □1轻 □2中 □3重 |
|  恶心或呕吐 | | □1轻 □2中 □3重 | 头昏 | □1轻 □2中 □3重 |
|  耳鸣或耳聋 | | □1轻 □2中 □3重 |  头痛 | □1轻 □2中 □3重 |
|  发热 | | □1轻 □2中 □3重 |  口疮 | □1轻 □2中 □3重 |
|  浮肿 | | □1轻 □2中 □3重 |  头胀 | □1轻 □2中 □3重 |
|  腹胀 | | □1轻 □2中 □3重 |  脘痞 | □1轻 □2中 □3重 |
|  咳嗽 | | □1轻 □2中 □3重 |  畏寒 | □1轻 □2中 □3重 |
|  咽痛 | | □1轻 □2中 □3重 |  胃痛 | □1轻 □2中 □3重 |
|  口干欲饮 | | □1轻 □2中 □3重 |  胁胀 | □1轻 □2中 □3重 |
| 烧心或反酸 | | □1轻 □2中 □3重 |  腰酸 | □1轻 □2中 □3重 |
|  肢凉 | | □1轻 □2中 □3重 |  肢麻 | □1轻 □2中 □3重 |
| 失眠 | | □1轻 □2中 □3重 |  |  |
| □ 其它兼症 | |  | | |
| 一般情况 | 纳食：□1正常 □2欠佳 □3很差 □4  睡眠：□1正常 □2难以入睡 □3睡后易醒 □4多梦 □5嗜睡 □6  大便：□1正常 □2便干便秘 □3大便偏稀 □4时干时稀 □5大便无力 □6  小便：□1正常 □2小便偏黄 □3小便清长 □4夜尿频 □5小便少 □6 | | | |

**舌脉诊：**

| 项目 | 具体征象 |
| --- | --- |
| 舌色 | □1淡红 □2淡白 □3红 □4绛 □5暗红 □6淡暗 □7暗紫 □8淡紫 □9青 □10青紫 □11瘀点 □12瘀斑 □13其他： |
| 苔色 | □1白 □2黄 □3灰 □4黑 □5其他： |
| 苔质 | □1薄 □2厚 □3滑 □4燥 □5糙 □6腻 □7腐 □8剥苔 □9其他： |
| 舌形 | □1老舌 □2嫩舌 □3胖大 □4齿痕 □5肿胀 □6瘦薄 □7点刺 □8裂纹 |
| 舌态 | □1歪斜 □2短缩 □3颤动 □4痿软 □强5硬 □6吐弄 □7其他： |
| 舌下  络脉 | □1正常 □2细短 □3色淡 □4粗胀 □5曲张 □6紫红 □7绛紫 □8其他： |
| 脉象 | □1沉 □2迟 □3数 □4细 □5弦 □6弱 □7滑 □8涩 □9促 □10结 □11代  □12其他： |

**处方：**

**访视2 研究期（_____月随访）**

**____年____月____日**

受试者末次给药时间： 年 月 日

治疗依从性： ％ [(处方量－未用量) / 处方量×100％]

受试者是否中止临床试验？ □_1_ 是；□_0_ 否

中止临床试验的日期：

中止临床试验的原因：□

1、不良事件(已填写不良事件表)

2、缺乏疗效

3、违背试验方案

4、失访

5、受试者不合作

6、出现其他并发症

7、其它(请祥述)

| 心动过缓病程（合计） | | | _________月 |
| --- | --- | --- | --- |
| Holter  检查记录 | □无  □有 _____次 | | |
|  | 心率： | 最慢心率_____，最快心率______，平均心率______  总心搏数_____，最长R-R间期_____s  停搏＞2s：____s，  心动过速（＞130bpm）有____跳，占总心率_____%  心动过缓（＜45bpm）有____跳，占总心率_____% | |
|  | 心率  变异 | SDNN_____ SDNN Idx_____ PNN50_____  rMSSD _____ SDANN ______ 三角指数 | |
|  | 室上性节律 | 总数____次，平均（小时）：  单发： 成对：  二联律： 三联律：  室上速：  最长室上速次数：  最大心率室上速 | |
|  | 室性  节律 | 总数____次，平均（小时）：  单发： 成对：  二联律： 三联律：  室速总数：_____阵 RonT：_____阵  最快心率的室速：  最小心率的室速 | |
|  | 房颤  分析 | 房颤心搏：  持续时间： 发生次数  ＞1.5s心搏有：____次  ＞2s心搏有：____次 | |
|  | 结论 |  | |

**中医主症：**

| 中医主症 | 记分 | 症状分级记分标准 |
| --- | --- | --- |
| 心 悸 |  | 0分：无。3分：偶发心悸，可自行缓解；6分：频繁发作，但能坚持工作。9分：心悸持续不解，影响生活和工作。 |
| 胸 闷 |  | 0分：无。3分：偶感胸闷，可自行缓解。6分：胸闷发作较频繁，但不影响正常生活和工作。9分：胸闷持续不解，影响生活和工作。 |
| 气 短 |  | 0分：无。2分：活动后气短。4分：稍动即气短。6分：平时亦感气短。 |
| 乏 力 |  | 0分：无。2分：重度活动感乏力；4分：中度活动感乏力。6分：轻度活动即乏力。 |
| 头 晕 |  | 0分：无。2分：偶感头晕，可自行缓解；4分：头晕发作较频繁，但不影响正常生活和工作。6分：头晕持续不解，影响生活和工作。 |
| 总分 |  | |

**中医兼症：**

|  | |  |  手足心热 | □_1_轻 □_2_中 □_3_重 |
| --- | --- | --- | --- | --- |
|  盗汗 | | □_1_轻 □_2_中 □_3_重 |  自汗 | □_1_轻 □_2_中 □_3_重 |
|  恶心或呕吐 | | □_1_轻 □_2_中 □_3_重 | 头昏 | □_1_轻 □_2_中 □_3_重 |
|  耳鸣或耳聋 | | □_1_轻 □_2_中 □_3_重 |  头痛 | □_1_轻 □_2_中 □_3_重 |
|  发热 | | □_1_轻 □_2_中 □_3_重 |  口疮 | □_1_轻 □_2_中 □_3_重 |
|  浮肿 | | □_1_轻 □_2_中 □_3_重 |  头胀 | □_1_轻 □_2_中 □_3_重 |
|  腹胀 | | □_1_轻 □_2_中 □_3_重 |  脘痞 | □_1_轻 □_2_中 □_3_重 |
|  咳嗽 | | □_1_轻 □_2_中 □_3_重 |  畏寒 | □_1_轻 □_2_中 □_3_重 |
|  咽痛 | | □_1_轻 □_2_中 □_3_重 |  胃痛 | □_1_轻 □_2_中 □_3_重 |
|  口干欲饮 | | □_1_轻 □_2_中 □_3_重 |  胁胀 | □_1_轻 □_2_中 □_3_重 |
| 烧心或反酸 | | □_1_轻 □_2_中 □_3_重 |  腰酸 | □_1_轻 □_2_中 □_3_重 |
|  肢凉 | | □_1_轻 □_2_中 □_3_重 |  肢麻 | □_1_轻 □_2_中 □_3_重 |
| 失眠 | | □_1_轻 □_2_中 □_3_重 |  |  |
| □ 其它兼症 | |  | | |
| 一般情况 | 纳食：□_1_正常 □_2_欠佳 □_3_很差 □_4_  睡眠：□_1_正常 □_2_难以入睡 □_3_睡后易醒 □_4_多梦 □_5_嗜睡 □_6_  大便：□_1_正常 □_2_便干便秘 □_3_大便偏稀 □_4_时干时稀 □_5_大便无力 □_6_  小便：□_1_正常 □_2_小便偏黄 □_3_小便清长 □_4_夜尿频 □_5_小便少 □_6_ | | | |

**舌脉诊：**

| 项目 | 具体征象 |
| --- | --- |
| 舌色 | □_1_淡红 □_2_淡白 □_3_红 □_4_绛 □_5_暗红 □_6_淡暗 □_7_暗紫 □_8_淡紫  □_9_青 □_10_青紫 □_11_瘀点 □_12_瘀斑 □_13_其他： |
| 苔色 | □_1_白 □_2_黄 □_3_灰 □_4_黑 □_5_其他： |
| 苔质 | □_1_薄 □_2_厚 □_3_滑 □_4_燥 □_5_糙 □_6_腻 □_7_腐 □_8_剥苔 □_9_其他： |
| 舌形 | □_1_老舌 □_2_嫩舌 □_3_胖大 □_4_齿痕 □_5_肿胀 □_6_瘦薄 □_7_点刺 □_8_裂纹 |
| 舌态 | □_1_歪斜 □_2_短缩 □_3_颤动 □_4_痿软 □强_5_硬 □_6_吐弄 □_7_其他： |
| 舌下络脉 | □_1_正常 □_2_细短 □_3_色淡 □_4_粗胀 □_5_曲张 □_6_紫红 □_7_绛紫 □_8_其他： |
| 脉象 | □_1_沉 □_2_迟 □_3_数 □_4_细 □_5_弦 □_6_弱 □_7_滑 □_8_涩 □_9_促 □_10_结 □_11_代□_12_其他： |

**处方：**

**访视3 研究期（_____月随访）**

**____年____月____日**

受试者末次给药时间： 年 月 日

治疗依从性： ％ [(处方量－未用量) / 处方量×100％]

受试者是否中止临床试验？ □_1_ 是；□_0_ 否

中止临床试验的日期：

中止临床试验的原因：□

1、不良事件(已填写不良事件表)

2、缺乏疗效

3、违背试验方案

4、失访

5、受试者不合作

6、出现其他并发症

7、其它(请祥述)

| 心动过缓病程（合计） | | | _________月 |
| --- | --- | --- | --- |
| Holter  检查记录 | □无  □有 _____次 | | |
|  | 心率： | 最慢心率_____，最快心率______，平均心率______  总心搏数_____，最长R-R间期_____s  停搏＞2s：____s，  心动过速（＞130bpm）有____跳，占总心率_____%  心动过缓（＜45bpm）有____跳，占总心率_____% | |
|  | 心率  变异 | SDNN_____ SDNN Idx_____ PNN50_____  rMSSD _____ SDANN ______ 三角指数 | |
|  | 室上性节律 | 总数____次，平均（小时）：  单发： 成对：  二联律： 三联律：  室上速：  最长室上速次数：  最大心率室上速 | |
|  | 室性  节律 | 总数____次，平均（小时）：  单发： 成对：  二联律： 三联律：  室速总数：_____阵 RonT：_____阵  最快心率的室速：  最小心率的室速 | |
|  | 房颤  分析 | 房颤心搏：  持续时间： 发生次数  ＞1.5s心搏有：____次  ＞2s心搏有：____次 | |
|  | 结论 |  | |

**中医主症：**

| 中医主症 | 记分 | 症状分级记分标准 |
| --- | --- | --- |
| 心 悸 |  | 0分：无。3分：偶发心悸，可自行缓解；6分：频繁发作，但能坚持工作。9分：心悸持续不解，影响生活和工作。 |
| 胸 闷 |  | 0分：无。3分：偶感胸闷，可自行缓解。6分：胸闷发作较频繁，但不影响正常生活和工作。9分：胸闷持续不解，影响生活和工作。 |
| 气 短 |  | 0分：无。2分：活动后气短。4分：稍动即气短。6分：平时亦感气短。 |
| 乏 力 |  | 0分：无。2分：重度活动感乏力；4分：中度活动感乏力。6分：轻度活动即乏力。 |
| 头 晕 |  | 0分：无。2分：偶感头晕，可自行缓解；4分：头晕发作较频繁，但不影响正常生活和工作。6分：头晕持续不解，影响生活和工作。 |
| 总分 |  | |

**中医兼症：**

|  | |  |  手足心热 | □_1_轻 □_2_中 □_3_重 |
| --- | --- | --- | --- | --- |
|  盗汗 | | □_1_轻 □_2_中 □_3_重 |  自汗 | □_1_轻 □_2_中 □_3_重 |
|  恶心或呕吐 | | □_1_轻 □_2_中 □_3_重 | 头昏 | □_1_轻 □_2_中 □_3_重 |
|  耳鸣或耳聋 | | □_1_轻 □_2_中 □_3_重 |  头痛 | □_1_轻 □_2_中 □_3_重 |
|  发热 | | □_1_轻 □_2_中 □_3_重 |  口疮 | □_1_轻 □_2_中 □_3_重 |
|  浮肿 | | □_1_轻 □_2_中 □_3_重 |  头胀 | □_1_轻 □_2_中 □_3_重 |
|  腹胀 | | □_1_轻 □_2_中 □_3_重 |  脘痞 | □_1_轻 □_2_中 □_3_重 |
|  咳嗽 | | □_1_轻 □_2_中 □_3_重 |  畏寒 | □_1_轻 □_2_中 □_3_重 |
|  咽痛 | | □_1_轻 □_2_中 □_3_重 |  胃痛 | □_1_轻 □_2_中 □_3_重 |
|  口干欲饮 | | □_1_轻 □_2_中 □_3_重 |  胁胀 | □_1_轻 □_2_中 □_3_重 |
| 烧心或反酸 | | □_1_轻 □_2_中 □_3_重 |  腰酸 | □_1_轻 □_2_中 □_3_重 |
|  肢凉 | | □_1_轻 □_2_中 □_3_重 |  肢麻 | □_1_轻 □_2_中 □_3_重 |
| 失眠 | | □_1_轻 □_2_中 □_3_重 |  |  |
| □ 其它兼症 | |  | | |
| 一般情况 | 纳食：□_1_正常 □_2_欠佳 □_3_很差 □_4_  睡眠：□_1_正常 □_2_难以入睡 □_3_睡后易醒 □_4_多梦 □_5_嗜睡 □_6_  大便：□_1_正常 □_2_便干便秘 □_3_大便偏稀 □_4_时干时稀 □_5_大便无力 □_6_  小便：□_1_正常 □_2_小便偏黄 □_3_小便清长 □_4_夜尿频 □_5_小便少 □_6_ | | | |

**舌脉诊：**

| 项目 | 具体征象 |
| --- | --- |
| 舌色 | □_1_淡红 □_2_淡白 □_3_红 □_4_绛 □_5_暗红 □_6_淡暗 □_7_暗紫 □_8_淡紫  □_9_青 □_10_青紫 □_11_瘀点 □_12_瘀斑 □_13_其他： |
| 苔色 | □_1_白 □_2_黄 □_3_灰 □_4_黑 □_5_其他： |
| 苔质 | □_1_薄 □_2_厚 □_3_滑 □_4_燥 □_5_糙 □_6_腻 □_7_腐 □_8_剥苔 □_9_其他： |
| 舌形 | □_1_老舌 □_2_嫩舌 □_3_胖大 □_4_齿痕 □_5_肿胀 □_6_瘦薄 □_7_点刺 □_8_裂纹 |
| 舌态 | □_1_歪斜 □_2_短缩 □_3_颤动 □_4_痿软 □强_5_硬 □_6_吐弄 □_7_其他： |
| 舌下络脉 | □_1_正常 □_2_细短 □_3_色淡 □_4_粗胀 □_5_曲张 □_6_紫红 □_7_绛紫 □_8_其他： |
| 脉象 | □_1_沉 □_2_迟 □_3_数 □_4_细 □_5_弦 □_6_弱 □_7_滑 □_8_涩 □_9_促 □_10_结 □_11_代□_12_其他： |

**处方：**
